# Effectiveness and safety of non-vitamin K antagonist oral anticoagulants in octogenarian patients with non-valvular atrial fibrillation

**DOI:** 10.1371/journal.pone.0211766

**Published:** 2019-03-07

**Authors:** Hyue Mee Kim, Eue-Keun Choi, Chan Soon Park, Myung-Jin Cha, Seo-Young Lee, Joon-Myung Kwon, Seil Oh

**Affiliations:** 1 Department of Internal Medicine, Seoul National University Hospital, Seoul, Republic of Korea; 2 Division of Cardiology, Heart Stroke Vascular Center, Mediplex Sejong Hospital, Incheon, Republic of Korea; 3 Department of Emergency medicine, Mediplex Sejong Hospital, Incheon, Republic of Korea; Maastricht University Medical Center, NETHERLANDS

## Abstract

**Background and objective:**

Elderly patients with atrial fibrillation (AF) are known to have a high risk of stroke and bleeding. We investigated the effectiveness and safety of non-vitamin K antagonist oral anticoagulants (NOACs) in octogenarian patients with non-valvular AF compared with warfarin.

**Methods:**

A total of 687 octogenarian patients with AF who were administered NOACs (n = 403) or warfarin (n = 284) for stroke prevention between 2012 and 2016 were included. Thromboembolic (TE) events (stroke or systemic embolism), major bleeding events, and all-cause death were analyzed.

**Results:**

The NOACs group (age 83.4±3.2 years, women 52.4%, CHA_2_DS_2_-VASc score 5.0±1.8) comprised 141 dabigatran, 158 rivaroxaban, and 104 apixaban users. Most patients from the NOACs group had been prescribed a reduced dose of medication (85.6%). During 14±18 months of follow-up periods, there were 19 TE events and 18 major bleeding events. Patients with NOAC showed a lower risk of TE (1.84 vs. 2.71 per 100 person-years, hazard ration [HR] 0.134, 95% confidence interval [CI] 0.038–0.479, P = 0.002), major bleeding (1.48 vs. 2.72 per 100 person-years, HR 0.110, 95% CI 0.024–0.493, P = 0.001), and all-cause death (2.57 vs. 3.50 per 100 person-years, HR 0.298, 95% CI 0.108–0.824, P = 0.020).

**Conclusion:**

In octogenarian Asian patients with AF, NOACs might be associated with lower risks of thromboembolic events, major bleeding, and all-cause death than warfarin. Although most patients had received reduced doses, on-label use of NOACs was effective and safe.

## Introduction

Atrial fibrillation (AF) is the most common arrhythmia observed in elderly patients, and one of the major causes of stroke in this population. The prevalence and incidence of AF in patients at a very advanced age (≥80 years) has shown a significant increase in Asians.[1,2] With the aging of the general population, the burden of AF and its complications has also been increasing. Elderly patients with AF are at an increased risk of both stroke and major bleeding. Although anticoagulation therapy using vitamin K antagonists reduces the risk of stroke in the elderly patients, warfarin has shown an increased risk of bleeding in the elderly population.[3,4] Additionally, warfarin use is associated with several limitations in elderly patients for the risk of cognitive impairment, poor medication adherence, and a narrow therapeutic range.[5–8] These limitations have been partly controlled by the introduction of non-vitamin K antagonist oral anticoagulants (NOACs), which have demonstrated comparable results in terms of stroke prevention and better safety outcomes in reducing the risk of major bleeding.[9–12] Recently, we reported the effectiveness and safety of NOACs in Asian patients with AF and showed that NOACs when compared to warfarin are associated with a comparable risk of stroke and a lower risk of bleeding and all-cause death.[13] However, there is a still lack of data regarding NOACs effectiveness and safety in patients at a very advanced age (≥80 years), particularly among Asians. Elderly patients (those in the 9^th^ decade) usually show a greater number of comorbidities and concomitant medication use, as well as a higher prevalence of impaired renal function than that observed in younger patients. Thus, effective and safe anticoagulation in octogenarian patients with AF is a critical issue. In this study, we sought to investigate the effectiveness and safety of NOACs in a real-world octogenarian patients with non-valvular AF.

## Methods

### Study population

We retrospectively analyzed a database of consecutive patients diagnosed with non-valvular AF who were older than 80 years old receiving warfarin or NOACs (dabigatran, rivaroxaban, and apixaban) at Seoul National University Hospital between January 2012 and June 2016. We focused on patients with non-valvular AF by excluding patients with rheumatic mitral valve disease or surgery for valve diseases. Patients were excluded if they had other diseases necessitating warfarin administration such as pulmonary embolism and deep vein thrombosis. After excluding 157 patients who were taking anticoagulants for conditions other than AF, 687 patients were deemed eligible for this study. Enrolled patients were divided into 2 groups: 403 patients were classified into the NOACs and 284 were classified into the warfarin group. Patients were assigned to each group when they received NOACs or warfarin for more than 1 day. Patients were censored when anticoagulant was changed to another or discontinued. Using drug labeling (S1 Table) as a guide for classification, patients administered NOACs were categorized based on those who received the on-label recommended dose, those who were prescribed with off-label NOACs (under-dosed or over-dosed). Clinical data regarding baseline characteristics were retrieved from patients’ medical records. The risk factors for thromboembolism were evaluated using the CHADS_2_ and the CHA_2_DS_2_-VASc score. Among the laboratory tests performed, serum levels of blood urea nitrogen, creatinine, and hemoglobin were measured at the time of enrollment. The estimated glomerular filtration rate (GFR) was calculated using the Modification of Diet in Renal Disease formula.[14] Our study was approved by the Seoul National University Hospital Institutional Review Board (1604-073-754) and was conducted in accordance with the Declaration of Helsinki. This study was retrospective cohort study, thus the informed consent was waived by the ethics committee.

### Study outcomes

Patients were followed in outpatient clinics, and their clinical records were reviewed. In the warfarin group, anticoagulation control was assessed using the time in therapeutic range (TTR), which was assessed by Rosendaal method.[15] The primary outcomes evaluating effectiveness and safety were the occurrence of ischemic stroke and/or systemic embolism and major bleeding, respectively. Major bleeding was defined as bleeding needed transfusion of at least 2 units of red blood cells, a drop in hemoglobin more than 2g/L, a surgical revision required due to bleeding, bleeding into critical sites (intracranial, intra-ocular, intra-articular, retroperitoneal, and overt gastrointestinal (GI) bleeding), or fatal bleeding. We also included all-cause deaths as an endpoint.

### Statistical analysis

Continuous variables were expressed as mean ± SD and categorical variables as percentages. A 2-sided P value <0.05 was considered statistically significant. Patient characteristics were compared between groups using a chi-squared test for categorical variables and the Student’s t test for continuous variables. Event rates were estimated using event counts and exposure following time. Univariate Cox proportional hazard regression analyses were performed to evaluate the predictive values of each variable, and variables observed to be significant were subjected to multivariable Cox proportional hazard regression model. We used stepwise backward elimination to select factors for inclusion in the multivariable analysis (inclusion criteria, P<0.05; exclusion criteria, P>0.1) because of the relatively small number of events (S2 Table). The hazard ratio (HR) and 95% confidence interval (CI) were calculated. Event-free survival analyses were conducted by Kaplan–Meier method with log-rank test and the Cox proportional hazard model. To estimate the effectiveness and safety of NOACs in the presence of confounding factors, weighted Kaplan-Meier analysis using inverse probability treatment weighting (IPTW) was performed. IPTW was based on the propensity score calculated with variables including age, sex, hypertension, diabetes mellitus, congestive heart failure, vascular disease, and stroke. All analyses were performed using the IBM SPSS statistics version 22 software (SPSS Inc., Chicago, IL, USA) and R version 3.4.3. (R Foundation for Statistical Computing).

## Results

### Baseline characteristics

The baseline demographic characteristics of all groups of patients are depicted in Table 1. A total of 687 patients were included in this study. 403 patients were administered NOACs and 284 were administered. The mean age was 83.4±3.1 years and 345 (50.2%) of the patients were women. No statistically significant differences were observed in terms of age, sex, and body mass index (BMI). The patients in the NOACs group showed higher CHADS_2_ score (2.8±1.3 vs. 2.0±0.8) and CHA_2_DS_2_-VASc score (5.0±1.8 vs. 3.5±1.0) and higher prevalence of comorbidities such as congestive heart failure (21.1% vs. 4.2%) previous stroke (35.2% vs. 8.8%). Patients from both groups contributed 786 patient-years of on-therapy follow-up. The median follow-up duration was 7.8 months (inter-quartile range [IQR]: 2.5–19.3 months), 5.5 months (IQR 1.8–8.9 months) for the NOACs and 15.3 months (IQR 4.0–42.6 months) for the warfarin group. The mean TTR of the warfarin group was 49%.

**Table 1:** Baseline characteristics.

	NOACs (n = 403)	WFR (n = 284)	P value †
Age, yrs	83.4±3.2	83.5±3.1	0.529
Female (%)	211 (52.4%)	134 (47.2%)	0.182
Height, cm	157.8±14.1	158.7±12.0	0.847
Body weight, kg	60.2±14.3	58.7±11.6	0.392
BMI (Kg/m^2^)	24.7±14.4	23.6±9.0	0.326
Past medical history			
Hypertension	246 (61.0%)	164 (57.7%)	0.386
Diabetes mellitus	124 (30.8%)	80 (28.2%)	0.463
Congestive heart failure	85 (21.1%)	12 (4.2%)	<0.001
Previous stroke	142 (35.2%)	25 (8.8%)	<0.001
Dementia	39 (9.7%)	16 (5.6%)	0.054
Lab			
Hemoglobin	12.9±1.7	12.9±1.9	0.706
BUN	18.9±7.7	21.0±9.8	0.007
Creatinine	1.1±0.3	1.2±0.8	0.004
GFR			
Normal (>80mL/min)	86 (24.7%)	49 (21.4%)	0.04
Mild impairment (50-80mL/min)	200 (57.5%)	119 (52.0%)	0.04
Moderate impairment (30-50mL/min)	56 (16.1%)	50 (21.8%)	0.04
Severe impairment (≤30mL/min)	6 (1.7%)	11 (4.8%)	0.04
CHADS_2_ score	2.8±1.3	2.0±0.8	<0.001
CHA_2_DS_2_-VASc	5.0±1.8	3.5±1.0	<0.001
HAS-BLED score	2.5±1.0	2.6±1.0	0.115
NOAC			
Regular dose	58 (14.4%)	-	
Reduced dose	345 (85.6%)	-	
Median F/U duration, months	5.5 (1.8–8.9)	15.3 (4.0–42.6)	<0.001

Values are presented as mean ± SD, as n (%), or median (interquartile range).

WFR = warfarin

^†^P value between NOACs and warfarin groups

### Thromboembolic and major bleeding events and all-cause deaths

**Fig 1:**
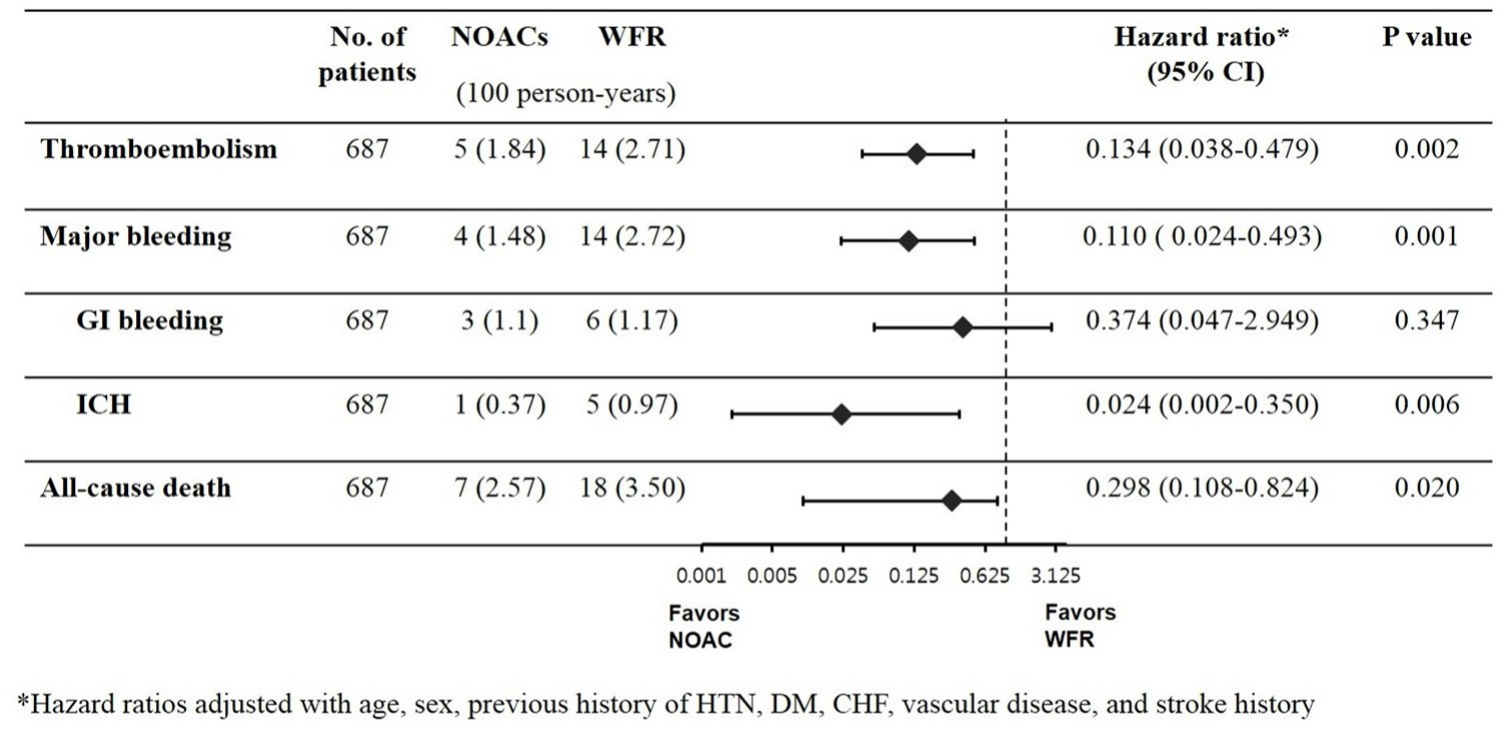
Clinical outcomes based on the treatment group. Hazard ratio adjusted with age, sex, previous history of hypertension, diabetes mellitus, congestive heart failure, vascular disease and stroke history. NOAC, non-vitamin K antagonist oral anticoagulants; WFR, warfarin; GI, gastrointestinal; ICH, intracranial hemorrhage.

During the follow-up, stroke or systemic embolic events were observed in 19 patients, and major bleeding events in 18. Fig 1 summarizes the clinical outcomes based on the treatment group. The overall incidence rate of thromboembolic events (TE) was 2.41 per 100 person-years. The overall incidence of major bleeding, GI bleeding, and intracranial hemorrhage (ICH) was 2.29, 1.15, and 0.76 per 100 person-years, respectively.

**Fig 2:**
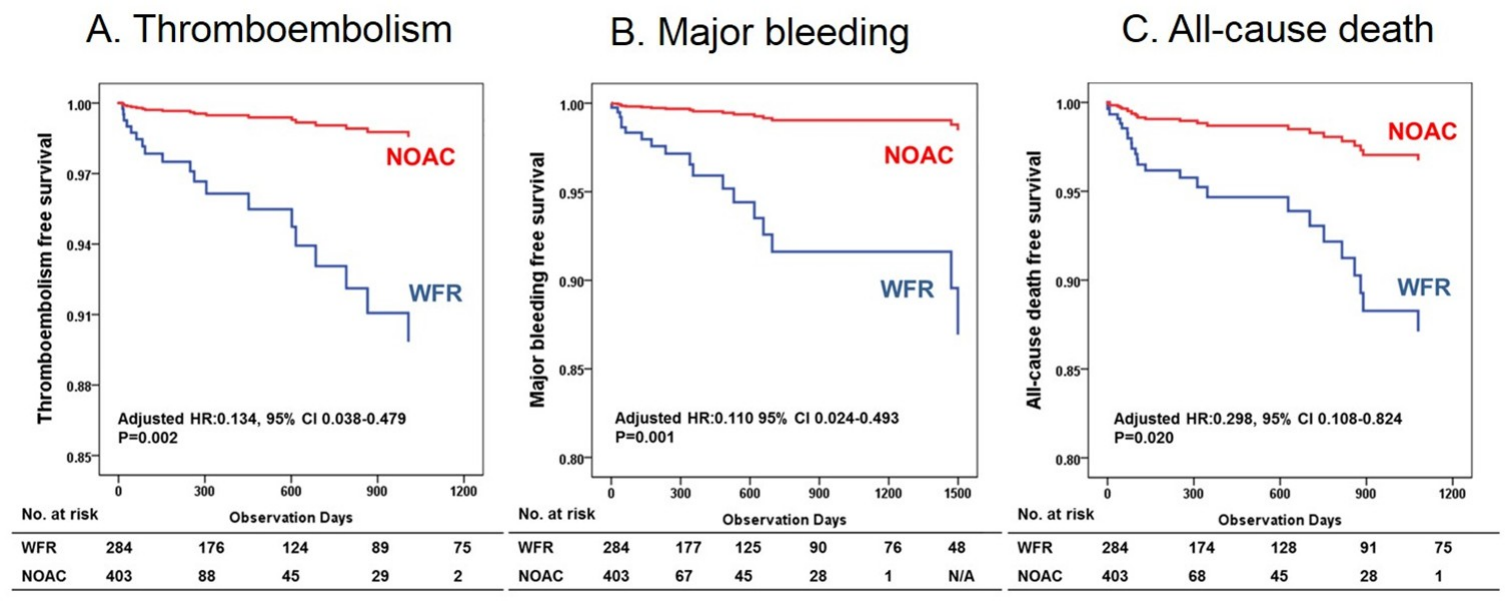
Risk-adjusted event-free survival according to the treatment. Event-free survival curves are demonstrated according to the treatment, adjusted for age, sex, previous history of hypertension, diabetes mellitus, congestive heart failure, vascular disease and stroke history. A) Event-free survival curve for thromboembolism, B) Event-free survival curve for major bleeding, C) Event-free survival curve for all-cause death. NOAC, non-vitamin K antagonist oral anticoagulants; WFR, warfarin.

A comparison between the NOACs and warfarin groups showed statistically significant differences in TE and major bleeding events and all-cause deaths after adjusting for potential confounders (Fig 2). Stroke or systemic embolism occurred in 5 patients in the NOACs and 14 patients in the warfarin group. In univariate comparisons, there was no significant difference in TE events between NOACs and warfarin group (HR 0.468, 95% CI 0.163–1.344, P = 0.158). After adjusting for multiple variables including age, sex, previous history of hypertension, diabetes, congestive heart failure, vascular disease, and previous stroke, the NOACs group showed a lower risk of TE than that observed in the warfarin group (HR 0.134, 95% CI 0.038–0.479, P = 0.002, Fig 2A).

There were 14 major bleeding episodes in the warfarin and 4 in the NOACs group. Multivariate analysis showed that the NOACs demonstrated a lower risk of major bleeding events than that observed in the warfarin group (HR 0.110, 95% CI 0.024–0.493, P = 0.001, Fig 2B). Among the major bleeding events, GI bleeding was the most common type (50.0%). The risk of major GI bleeding was similar between the NOACs and the warfarin group (HR 0.374, 95% CI 0.047–2.949, P = 0.347). ICH occurred in 5 patients in the warfarin group, whereas there was only 1 event in the NOACs group. Multivariate analysis revealed that the NOACs demonstrated a lower risk of ICH than that observed in the warfarin group (HR 0.024, 95% CI 0.002–0.350, P = 0.006).

In addition, the weighted Kaplan-Meier analysis using IPTW consistently revealed lower risks of thromboembolism (HR 0.131, 95% CI 0.046–0.371, P = 0.0023) and major bleeding (HR 0.259, 95% CI 0.062–1.087, P = 0.051) in the patients with NOACs (S1 Fig and S3 Table).

Among patients in whom data regarding GFR were available, 76% showed more than mild chronic kidney disease (CKD). Two-thirds of TE events (11 of 17, 65%) and major bleeding events (10 of 17, 62.5%) occurred in those who have more than mild CKD. No TE and major bleeding event was observed in the NOACs group with moderate to severe CKD, whereas 3 TE and 4 major bleeding events were observed in the warfarin group with moderate to severe CKD. NOACs and warfarin did not demonstrate a statistically significant effect on the occurrence of TE and major bleeding events based on renal function (P for interaction = 0.579 and 0.288, respectively).

Eighteen patients died in the warfarin group and 7 in the NOACs group (3.5 per 100 person-years, vs. 2.57 per 100 person-years, respectively). All-cause deaths were also observed to be significantly lower in the NOACs group (HR 0.298, 95% CI 0.108–0.824, P = 0.020, Fig 2C). Among the patients who died, 4 died after TE and 3 after major bleeding events.

### Clinical outcomes based on the types and doses of NOACs

Among the 403 patients in the NOACs group, 141 received dabigatran, 158 received rivaroxaban, and 104 received apixaban. Among the patients who developed TE events, 3 had received dabigatran, 1 had received rivaroxaban, and 1 had received apixaban, respectively (2.38 vs. 1.15 vs. 1.72 per 100 person-years, respectively). Among patients with major bleeding events in the NOACs group, 2 had been treated with dabigatran, 1 with rivaroxaban, and 1 with apixaban, respectively (1.60 vs. 1.15 vs. 1.72 per 100 person-years, respectively).

**Fig 3:**
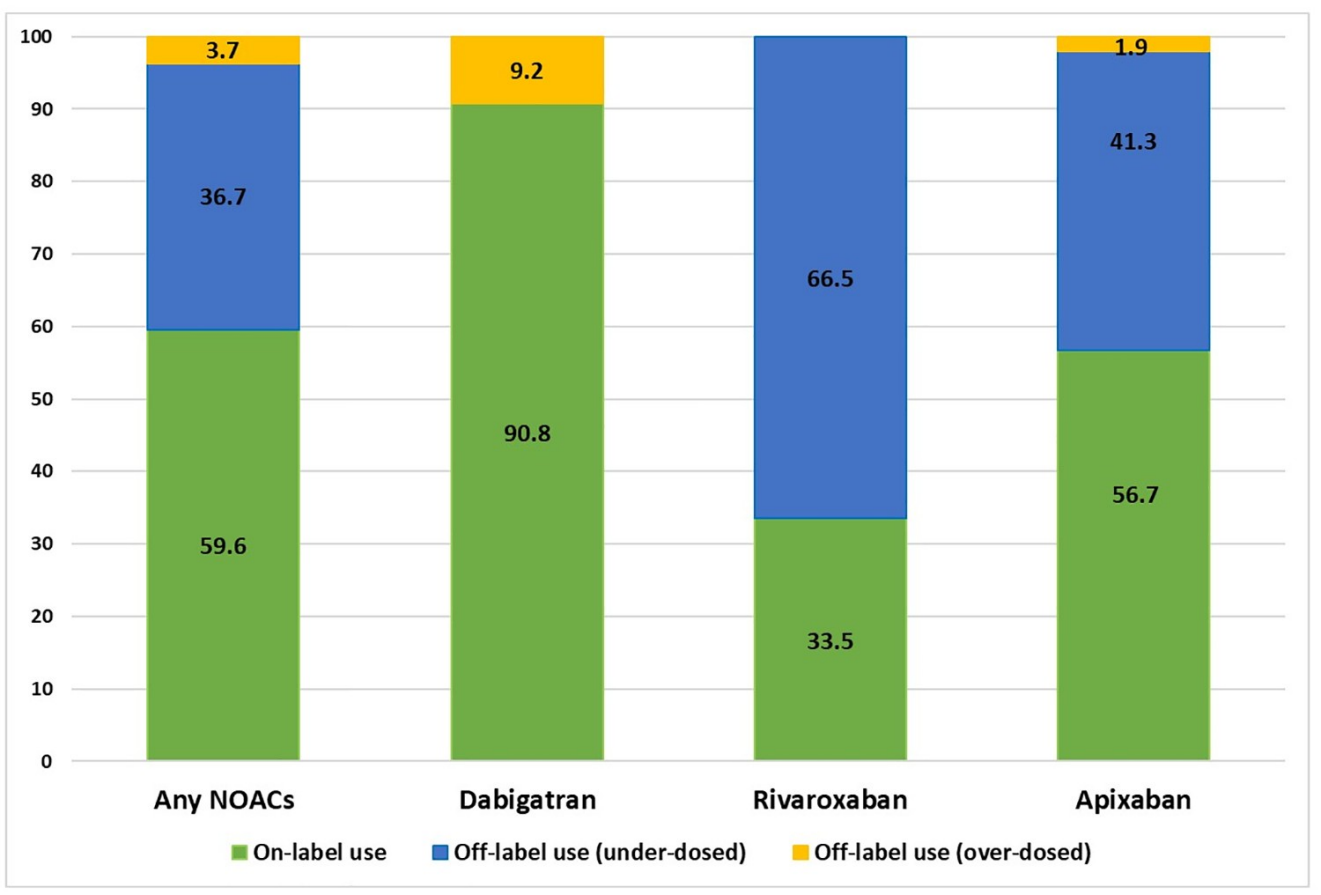
Proportion of NOACs dosing according to label.

**Fig 4:**
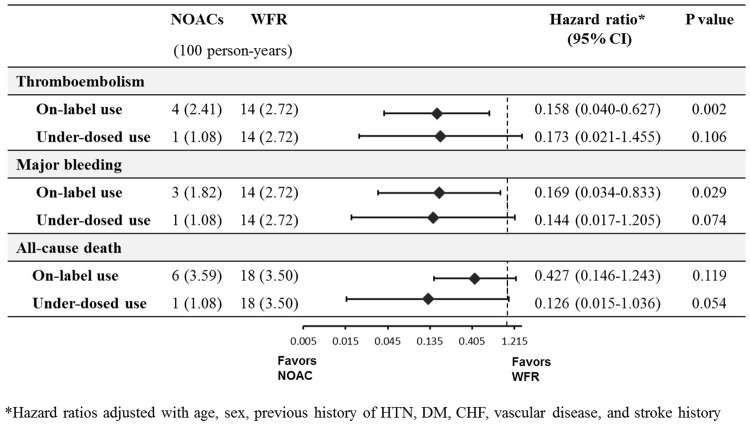
Clinical outcomes according to dose of NOACs. Hazard ratio adjusted with age, sex, previous history of hypertension, diabetes mellitus, congestive heart failure, vascular disease and stroke history. NOAC, non-vitamin K antagonist oral anticoagulants; WFR, warfarin.

In this study, most of patients (n = 345, 85.6%) had been prescribed a reduced dose (128 patients received dabigatran at a dose of 110 mg twice daily, 36 patients received rivaroxaban at a dose of 10 mg daily, 88 patients received rivaroxaban at a dose of 15 mg daily, and 93 patients received apixaban at a dose of 2.5 mg twice daily), There was no significant difference between two groups except for renal dysfunction (S4 Table). All clinical events occurred in those prescribed reduced doses of NOACs. After adjusting for covariables, patients who received reduced doses of NOACs showed a lower risk of TE events (HR 0.151, 95% CI 0.042–0.548, P = 0.004), major bleeding events (HR 0.125, 95% CI 0.027–0.586, P = 0.008), and all-cause deaths (HR 0.277, 95% CI 0.089–0.865, P = 0.27) compared to those who received warfarin. Based on dosage recommendations, 240 (59.6%) patients were prescribed the recommended dose of NOACs, whereas 36.7% were categorized as under-dosed and 3.7% as over-dosed (Fig 3). Most patients administered dabigatran were prescribed the drug based on recommended dose, whereas 67% of patients administered rivaroxaban were categorized as under-dosed. When comparing the patient groups with on-label and off-label under-dosed NOACs separately, significantly lower incidence rates of TE (HR 0.158, 95% CI 0.040–0.627, P = 0.002) and major bleeding events (HR 0.169, 95% CI 0.034–0.833 P = 0.029) were observed in the on-label NOACs than that observed in the warfarin group (Fig 4). Moreover, the patients with off-label under-dosed NOACs use showed similar trend in TE, major bleeding and all-cause death compared with those with warfarin.

## Discussion

The main findings of this study are: (1) NOACs showed a lower risk of TE, major bleeding, and all-cause deaths than that observed with warfarin in octogenarian patients with non-valvular AF, (2) when comparing between the on- and off-label use of NOACs, the former showed better effectiveness and safety compared with warfarin in octogenarian patients, and (3) most octogenarian patients with AF showed renal dysfunction, but the effectiveness and safety of NOACs showed results that were comparable to those observed with warfarin use in these patients.

Several studies have reported the safety and effectiveness of NOACs in elderly patients. Most of them have been subgroup analysis or meta-analysis of representative randomized clinical trials assessing these drugs. Table 2 summarizes the studies reporting the effectiveness and safety of NOACs in octogenarian patients with AF in a real-world setting.[16–21] A recent European study has reported that 3 NOACs mentioned in this study showed a similar risk of TE and major bleeding events compared to warfarin in patients aged more than 80 years.[18] In an elderly Asian elderly population, a few studies have reported the effectiveness and safety of NOACs in octogenarian patients with AF.[19,20] However, the number of patients investigated in these previous studies involving Asian patients was inadequate to conclusively establish the difference between the role of NOACs and warfarin in stroke prevention and reduction in major bleeding events. We observed that NOACs showed better effectiveness in TE prevention and a reduced risk of major bleeding, particularly in ICH compared to that observed with warfarin in octogenarian patients with AF. Furthermore, there was mortality benefit in NOACs group compared to warfarin. In contrast to previous Asian studies, 3 NOACs (dabigatran, rivaroxaban, and apixaban) were included and analyzed in this study.

**Table 2:** Comparisons of previous studies for the effectiveness and safety of NOACs in elderly patients compared with warfarin.

	Population	NOACs included in the study	Effectiveness (stroke prevention)	Safety		All-cause death
				Major bleeding	ICH or GI bleeding	
Halvorsen S et al. (2014)[17]	≥75 years (subgroup, n = 5,678) European (40.3%), American (43.8%), Asian (16.0%)	Apixaban	Reduced	Reduced	Reduced risk of ICH	Similar
Halperin JL et al. (2014)[21]	≥75 years (subgroup, n = 6229) European (53%), American (32%), Asian (15%)	Ribaroxaban	Similar	Similar	Similar risks for ICH Increased risk of GI bleeding	
Graham DJ et al. (2015)[16]	≥65 years, n = 134,414 American	Dabigatran	Reduced		Reduced risk of ICH Increased risk of GI bleeding	Reduced
Kwon CH et al. (2016)[19]	≥80 years, n = 293 Asian (Korea)	Dabigatran Rivaroxaban	Similar	Similar		
Chan PH et al. (2016)[20]	≥80 years, n = 571 Asian (China)	Dabigatran	Reduced		Similar risks for ICH	
Forslund T et al. (2017)[18]	≥80 years (subgroup, n = 7152) European (Sweden)	Dabigatran Rivaroxaban Apixaban	Similar	Similar	Increased risk of GI bleeding in regular dose	Similar
Current study	≥80 years, n = 687 Asian (Korea)	Dabigatran Rivaroxaban Apixaban	Reduced	Reduced	Reduced risk of ICH Similar risk of GI bleeding	Reduced

Although stroke prevention effect of NOACs in elderly patients have been reported to be comparable with that of warfarin, reports describing their effect on bleeding complications have been inconsistent. In major phase 3 clinical trials, although NOAC use was associated with a reduced risk of major bleeding events, particularly for ICH, the risk of GI bleeding was noted to be increased. A meta-analysis of randomized clinical trials showed that NOACs (including dabigatran, rivaroxaban, and apixaban) did not cause excessive bleeding in those aged >75 years.[22] Additionally, both dabigatran and rivaroxaban showed similar bleeding event rates compared to warfarin in octogenarian patients with AF in a real-world retrospective study.[16,19,20]. However, Halvorsen et al. reported that apixaban was shown to be superior to warfarin with respect to bleeding complications in patients aged >80 years.[17] They assessed the efficacy and safety of apixaban according to age as subgroup analysis performed in the Apixaban for Reduction in Stroke and Other Thromboembolic Events in Atrial Fibrillation (ARISTOTLE) trial. In the present study, patients treated with NOACs showed a lower risk of major bleeding events—ICH particularly was observed to be lower in the NOAC than the warfarin group, whereas the risk of GI bleeding was not statistically significantly different between the groups. The number of patients included in the study and the type of NOACs administered might explain the differences between studies in terms of outcomes.

In a post hoc analysis of bleeding events, dabigatran and rivaroxaban showed a higher event rate of GI bleeding in patients administered NOACs. Dabigatran was reported to show an interaction with a patient’s age for major and extracranial bleeding, whereas the risk of intracranial bleeding was consistently lower compared to that of warfarin irrespective of age.[23] Moreover, another study reported that dabigatran use was associated with an increased risk of major GI bleeding in women aged ≥75 years and men aged ≥85 years.[16] However, this result was most pronounced in patients treated with dabigatran at a dose of 150 mg twice daily, whereas in those treated with a reduced dose (75 mg twice daily) the risk of major bleeding events was similar to that of warfarin except for a lower risk of ICH associated with dabigatran use. Rivaroxaban use did not show a statistically significant difference in the occurrence of major bleeding events; however, the risk of GI bleeding was higher, particularly among elderly patients.[21] In this study, we observed that there was no statistically significant difference in major GI bleeding events between the NOAC and the warfarin groups, which could possibly be explained by the higher percentage of reduced dose prescriptions noted in this study, particularly those of rivaroxaban.

It is known that in real-world clinical practice, octogenarian patients are expected to show a higher risk of bleeding events and therefore tend to be prescribed reduced-dose NOACs. In this study, only 60% of patients received on-label dosing and approximately 40% received under-dosed NOACs. However, patients treated with on-label NOACs showed a lower risk of TE and major bleeding events than those observed with warfarin use suggesting that on-label use of NOACs would be effective and safe in octogenarian patients. Furthermore, reduced off-label doses (underdosing), does not guarantee a lower risk of bleeding events in patients at a very advanced age. Therefore, labeled prescription of NOAC would be recommended in octogenarian patients with AF.

The clinical outcomes of the present study were influenced by several factors. Firstly, in this study, patients belonging to the NOACs group showed significantly higher CHADS_2_ and CHA_2_DS_2_-VASc scores, which means their risk of developing TE events was higher with the use of NOACs. In univariate analysis, the effectiveness and safety outcomes were not significantly different between the groups. After adjusting for possible confounders, NOACs when compared with warfarin were associated with a reduced risk of TE, major bleeding events, and all-cause death with the difference being statistically significant. Considering high comorbidities in octogenarian patients, NOACs would be preferred to more effectively prevent TE and reduce the risk of major bleeding events. Secondly, elderly patients using warfarin have difficulty with maintaining a high TTR. Previous studies demonstrated poor TTR among patients using warfarin to prevent stroke in Asians.[24,25] That is because there were concerns of cerebral hemorrhage due to several reports describing that the incidence of hemorrhagic stroke was higher in Asians than in the western population.[26,27] Additionally, the recommended target international normalized ratio (INR) in those aged >70 years is 1.6–2.6 in Japan [28]; thus, a few clinicians in Korea tend to maintain the INR at values <2.0–3.0.

There are several limitations of the present study. First, this was a retrospective single-center study, thus the decision regarding the types and dose of anticoagulants used was not randomized. Therefore, residual confounding factors not adjusted for in the analysis could have affected our results. Furthermore, the reason why physicians had selected the underdosed NOAC is unclear. In ORBIT-AF II (Outcomes Registry for Better Informed Treatment of Atrial Fibrillation AF II) registry reporting that 9% of patients with AF had received off-label underdosed NOAC according to U.S labelling, the reason of underdosing is also unclear.[14] We speculate that physicians seem to consider the risk of bleeding more important in these fragile patients, which resulted to increase number of patients with underdosing. Second, the event rate was observed to be very low in the NOACs group; thus, the multivariate analysis could be over-fitted. To mitigate this problem, we used the stepwise backward elimination method to select factors for inclusion in the multivariate analysis and weighted Kaplan-Meier analysis using IPTW. However, the problem of overfitting was not completely solved by this statistical method, so the results of this study would be considered as suggestive. Although it would be reasonable to compare the outcomes between the groups using propensity score matching methods, the number of octogenarian patients was not adequate to perform this analysis. Also, we included 3 NOACs in this study; however, the number of each NOAC was not adequate for a comparison with warfarin respectively. Finally, the TTR in our warfarin group was low as in previous papers in Asian patients.[29–31] A recent retrospective study reported a mean TTR as 50% in Korean AF patients.[32] The poor TTR control in warfarin group might have affected the patients’ worse outcomes. However, this result also supports the clinical usefulness of NOACs in elderly patients.

## Conclusion

In octogenarian patients with AF, NOACs showed better outcomes in reducing the risk of TE, major bleeding events, and all-cause deaths when compared with warfarin. Although most patients had been prescribed a reduced dose, on-label use of NOACs was effective and safe in this patient population. It would be reasonable to prescribe NOACs rather than warfarin in octogenarian patients with non-valvular AF.

## Supporting information

S1 Fig Weighted Kaplan-Meier with use of inverse probability weighting. (TIF)

S1 Table Dosing criteria of NOACs. (DOCX)

S2 Table Multivariable analysis. (DOCX)

S3 Table Data adjusted with the use of inverse probability weighting. (DOCX)

S4 Table Baseline characteristics according to doses of NOACs. (DOCX)
