# Supplementary figures and images for: Effectiveness and safety of non-vitamin K antagonist oral anticoagulants in octogenarian patients with non-valvular atrial fibrillation
Source: PLoS One. 2019 Mar 7;14(3):e0211766. doi: 10.1371/journal.pone.0211766 (PMC6405244; doi:10.1371/journal.pone.0211766)

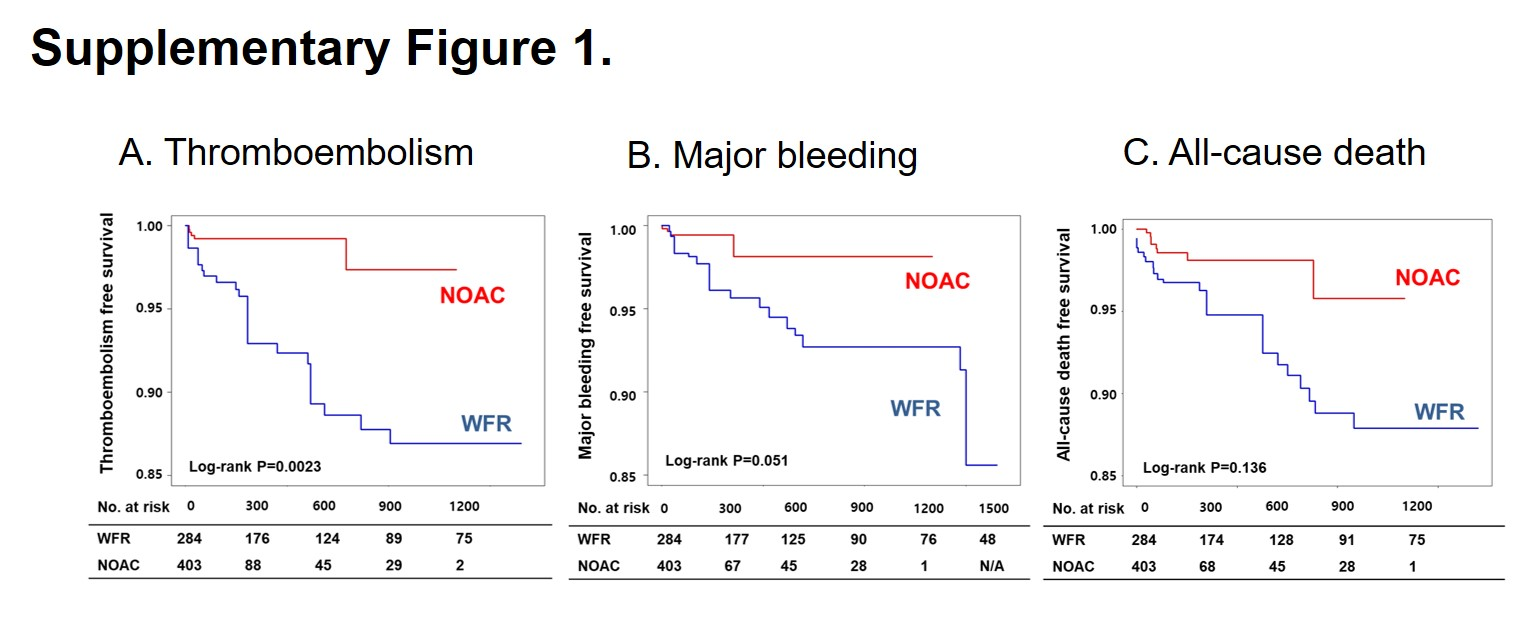

Supplement: S1 Fig — (TIF) [file pone.0211766.s001.tif]
