# Supplementary material for: Effectiveness and safety of non-vitamin K antagonist oral anticoagulants in octogenarian patients with non-valvular atrial fibrillation
Source: PLoS One. 2019 Mar 7;14(3):e0211766. doi: 10.1371/journal.pone.0211766 (PMC6405244; doi:10.1371/journal.pone.0211766)
Supplement: S1 Table — (DOCX) [file pone.0211766.s002.docx]

**S1 Table**. Dosing criteria of NOACs

| Dabigatran^1)^ |
| --- |
| - 150mg twice daily is standard dose - Patients aged 80 years or above should be treated with 110mg twice daily - Patients who receive verapamil should be reduced to 110mg twice daily - For patients with moderate renal impairment (CrCl 30-50mL/min), the recommended dose is 150mg twice daily. However, for patients with high risk of bleeding, a dose reduction of Pradaxa to 220 mg taken as one 110 mg capsule twice daily should be considered. - Patients with severe renal impairment (CrCl <30mL/min): not recommended |
| Rivaroxaban^2)^ |
| - 20mg once daily is standard dose - Patients with moderate or severe renal impairment (CrCl 15-49mL/min) : 15mg once daily - Patients with severe renal impairment (CrCl <15mL/min): not recommended |
| Apixaban^3)^ |
| - 5 mg twice daily unless patient has any 2 of the following:   - Age ≥80 years,   - body weight ≤60 kg,   - serum creatinine ≥1.5 mg/dL   then, reduce dose to 2.5 mg twice daily. |

**References**

1. Boehringer-Ingelheim. Dabigatran Prescribing Information. Available at:

<https://products.boehringer-ingelheim.com/pradaxa/sites/default/files/Prescriber_guide.pdf>

1. Bayer. Xarelto prescribing Information. Available at:

<https://www.xarelto.com/static/media/pdf/Xarelto_Prescriber_Guide.pdf>

1. Pfizer. Eliquis prescribing Information. Available at:

https://packageinserts.bms.com/pi/pi_eliquis.pdf
