# Supplementary material for: Effectiveness and safety of non-vitamin K antagonist oral anticoagulants in octogenarian patients with non-valvular atrial fibrillation
Source: PLoS One. 2019 Mar 7;14(3):e0211766. doi: 10.1371/journal.pone.0211766 (PMC6405244; doi:10.1371/journal.pone.0211766)
Supplement: S2 Table — (DOCX) [file pone.0211766.s003.docx]

**S2 Table. Multivariable analysis**

|  | **Multivariate analysis** | | | | |
| --- | --- | --- | --- | --- | --- |
|  | **HR** | **95% CI** | **P value** | **Regression coefficients** | **Standard errors** |
| **Thromboembolism** | | | | | |
| NOAC | 0.134 | 0.038-0.479 | 0.002 | -2.008 | 0.649 |
| Diabetes mellitus | 2.989 | 1.174-7.611 | 0.022 | 1.095 | 0.477 |
| CHF | 5.661 | 1.356-23.625 | 0.017 | 1.734 | 0.729 |
| Stroke | 6.129 | 2.212-16.980 | 0.000 | 1.813 | 0.520 |
| **Major Bleeding** | | | | | |
| NOAC | 0.110 | 0.024-0.493 | 0.001 | -2.210 | 0.766 |
| Age | 1.143 | 1.011-0.024 | 0.033 | 0.134 | 0.063 |
| CHF | 5.689 | 1.258-25.734 | 0.024 | 1.739 | 0.770 |
| Stroke | 6.871 | 2.381-19.824 | 0.000 | 1.927 | 0.541 |
| **All-cause death** | | | | | |
| NOAC | 0.298 | 0.108-0.824 | 0.020 | -1.210 | 0.519 |
| Age | 1.129 | 1.020-1.251 | 0.019 | 0.122 | 0.052 |
| Female | 0.506 | 0.218-1.175 | 0.113 | -0.681 | 0.430 |
| Stroke | 3.395 | 1.296-8.896 | 0.013 | 1.222 | 0.491 |
