# Supplementary material for: Effectiveness and safety of non-vitamin K antagonist oral anticoagulants in octogenarian patients with non-valvular atrial fibrillation
Source: PLoS One. 2019 Mar 7;14(3):e0211766. doi: 10.1371/journal.pone.0211766 (PMC6405244; doi:10.1371/journal.pone.0211766)
Supplement: S3 Table — (DOCX) [file pone.0211766.s004.docx]

**S3 Table.** Data adjusted with the Use of Inverse Probability Weighting

|  | Unadjusted Data | | | Data adjusted with IPTW | | |
| --- | --- | --- | --- | --- | --- | --- |
|  | NOACs  (n=403) | WFR  (n=284) | ASD | NOACs  (n=403) | WFR  (n=284) | ASD |
| Age, yrs | 83.4±3.1 | 83.2±3.0 | -0.0165 | 83.4±3.2 | 83.5±3.1 | -0.0028 |
| Female (%) | 52.4% | 47.2% | -0.0278 | 50.1% | 49.3% | 0.0031 |
| Past medical history |  |  |  |  |  |  |
| Hypertension | 61.0% | 57.7% | -0.0725 | 59.0% | 55.6% | -0.0018 |
| Diabetes mellitus | 30.8% | 28.2% | 0.29 | 29.4% | 29.1% | -0.0011 |
| Congestive Heart failure | 21.1% | 4.2% | 0.0232 | 13.5% | 11.3% | -0.0035 |
| Vascular Disease | 9.4% | 6.7% | 0.0771 | 8.2% | 7.6% | -0.0073 |
| Previous stroke | 35.2% | 8.8% | 0.183 | 24.3% | 21.6% | -0.002 |

WFR=warfarin; ASD = absolute standardized difference
