# Supplementary material for: Effectiveness and safety of non-vitamin K antagonist oral anticoagulants in octogenarian patients with non-valvular atrial fibrillation
Source: PLoS One. 2019 Mar 7;14(3):e0211766. doi: 10.1371/journal.pone.0211766 (PMC6405244; doi:10.1371/journal.pone.0211766)
Supplement: S4 Table — (DOCX) [file pone.0211766.s005.docx]

**S4 Table**. Baseline characteristics according to doses of NOACs

|  | Regular dose  (n=58) | Reduced dose  (n=345) | P value † |
| --- | --- | --- | --- |
| Age, yrs | 82.8±2.5 | 83.5±3.3 | 0.079 |
| Female (%) | 31 (53.4%) | 180 (52.2%) | 0.857 |
| Height, cm | 159.4±8.4 | 158.2±13.1 | 0.561 |
| Body weight, kg | 62.2±10.1 | 59.2±11.1 | 0.089 |
| BMI (Kg/m^2^) | 24.5±4.1 | 24.7±15.5 | 0.934 |
| Past medical history |  |  |  |
| Hypertension | 35 (60.3%) | 211 (61.2%) | 0.906 |
| Diabetes mellitus | 20 (34.5%) | 104 (30.1%) | 0.508 |
| Congestive heart failure | 8 (13.8%) | 77 (22.3%) | 0.141 |
| Previous stroke | 12 (39.7%) | 119 (34.5%) | 0.446 |
| Lab |  |  |  |
| Hemoglobin | 13.4±1.5 | 12.8±1.7 | 0.049 |
| BUN | 17.1±5.7 | 19.1±738 | 0.036 |
| Creatinine | 0.9±0.2 | 1.1±0.3 | 0.004 |
| GFR |  |  |  |
| Normal (>80mL/min) | 11 (22.9%) | 75 (25.0%) | 0.041 |
| Mild impairment (50-80mL/min) | 35 (72.9%) | 165 (55.0%) | 0.041 |
| Moderate impairment (30-50mL/min) | 2 (17.5%) | 54 (18.0%) | 0.041 |
| Severe impairment (≤30mL/min) | 0 (0%) | 6 (2.0%) | 0.041 |
| CHADS_2_ score | 2.8±1.3 | 2.8±1.3 | 0.821 |
| CHA_2_DS_2_-VASc | 5.1±1.8 | 4.9±1.8 | 0.638 |
| HAS-BLED score | 2.5±1.0 | 2.5±1.0 | 0.910 |

Values are presented as mean ± SD or as n (%).

 †P value between regular dose and reduced dose of NOACs
